# Comparison between the IT-MAIS and MUSS questionnaires with video-recording for evaluation of children who may receive a cochlear implantation

**DOI:** 10.1016/S1808-8694(15)30757-6

**Published:** 2015-10-19

**Authors:** Elaine Soares Monteiro Pinto, Cristina Broglia de Feitosa Lacerda, Paulo Rogério Catanhede Porto

**Affiliations:** 1 Master's degree in Human Communication Disorders - UNIFESP, professor at UNIMEP; 2 Post-doctoral course, CNR - Italy. Professor at the Piracicaba Methodist University; 3 Master's degree in Otorhinolaryngology/ UNICAMP, Otorhinolaryngologist, responsible for the Cochlear Implant Sector, Clinical Hospital, UNICAMP

**Keywords:** potential users, cochlear implant, direct observation, protocols, muss questionnaire

## Abstract

There is a great difficulty in determining earlier on which children would benefit or not from cochlear implants, especially because of their young age, the responses they give are very subtle.

**Aim:**

To compare results obtained through video-recording of the interactions of children who may receive a cochlear implant with the results obtained through evaluation protocols.

**Method:**

Seven children, with an average age of 39.7 months, with profound hearing loss were selected for the study. IT-MAIS and MUSS questionnaires were given to their parents/guardians of these children and the results were compared with the observation of the video-recordings.

**Results:**

It was possible to observe that the data is compatible with the auditory stages. However, the MUSS questionnaire data gathered during playful activities is very different. The questionnaire only takes into consideration the use of verbal language and therefore the majority of the evaluated children inevitably score low.

**Conclusion:**

Observing children play allows us to trace a better profile of linguistic behavior and aspects relative to language, that may presented differences in the questionnaire.

## INTRODUCTION

The diagnosis of hearing loss is being made earlier as a result of neonatal auditory surveillance programs. Yoshinaga-Itano (2003)^1^ has shown that children diagnosed early, and that received adequate interventions by age six months, develop language capabilities at the same rate as normal-hearing children of the same age, regardless of the degree of hearing loss. The use of hearing aids in children is frequently initiated before age six months, thanks to this process; it has also become possible to promptly identify those children that do not benefit from traditional sound amplification devices.

Conventional hearing aids are effective in treating hearing loss, but require sufficient cochlear reserve for good sound and speech perception, as these devices are no more than sound amplifiers. Bento et al. (2004)^2^ has stated that patients not benefiting from hearing aids are candidates for a second alternative for rehabilitating their hearing, namely cochlear implants.

Technological developments in medicine have made it possible to use cochlear implants in children; this has become an established technology for providing the hard of hearing with access to auditory signals that were previously not available when using traditional amplification aids. The US Food and Drug Administration (regulating agency for human and health services) recommends cochlear implants after age 12 months. Innovations in cochlear implants have provided not only improved tone thresholds, but also originated flexible devices that make it possible to use multiple speech sound digital processing strategies, which improve the patient's discrimination ability, according to Frederigue (2003).^3^

A cochlear implant is an induction coil - an internal receptor - that is implanted under the skin a little behind and above the ear, and a platinum wire - an active electrode - that is introduced into the cochlea. This wire contains minute electrodes that are implanted in the tympanic canal of cochlea to electrically stimulate nervous fibers in various positions along the cochlear spiral (Kandel, 2003).^4^

Cochlear implants, thus, partially take on cochlear functions, transforming sound energy into electrical signals (Ferrari et al., 2004).^5^

A child is considered for a cochlear implant when hearing aids yield no satisfactory gain in speech perception. According to Miyamoto et al. (2003),^6^ the gap between linguistic and chronological age should be minimized and auditory information should be introduced during critical language development periods to attain the benefits of early implants. These authors point out that early cochlear implant placement minimizes delayed language acquisition and fosters the development of appropriate language abilities according to the child's age. Language development benefits should be well defined and balanced against the potential risk associated with anesthesia and surgery at this age. Quittner et al. (2004)^7^ have stated that early cochlear implant placement - before structural and functional changes in the brain - is important for healthy development, enabling “normal” development, according to this author. Pulsifer et. al.'s (2003)^8^ papers suggest that cochlear implants may be beneficial for profoundly hearing-impaired children, resulting in improved sound detection, speech perception and global development one year after surgery, particularly in communication and social abilities; also, younger children tend to show more gains.

There are, however, significant difficulties in defining at an early age which children will benefit or not from cochlear implants; the responses at this young age are very subtle. Objective evaluation methods are preferred in the literature, usually composed of language assessment and speech perception protocols that yield quantitative measurements of performance; examples are the papers published by Bevilacqua et al. (1997),^9^ Svirsky et al. (2000),^10^ Pulsifer et al. (2003),^8^ Myamoto et al. (2003),^6^ and Ouellet et al. (2001).^11^ Many questionnaires on language development have been applied to parents in an attempt to learn more about the child's performance in this area, and thus, to verify the pertinence or indication of placing a cochlear implant. Frequently, however, parents that respond reveal imprecise knowledge about the true development of their children, a factor that may complicate any assessment based on these protocols. The MUSS (Meaningful Use of Speech Scale), which contains questions about oral language development, and the IT-MAIS (Infant Toddler - Meaningful Auditory Integration Scale), which focuses on the development of auditory stages, are used in many Brazilian health services.

The MUSS is a questionnaire developed by Robins and Osberger (1990),^12^ using day-to-day speech as synonymous with oral language. Nascimento (1997)^13^ adapted this scale for Brazil to characterize speech production in normal-hearing children. It was applied to a group of 15 parents of children aged between 2 and 5 years with normal neurological, otorhinolaryngological and audiological assessments and no history of communication or hearing disorders. The author concluded that the maximum score in the scale was reached at age 51 months, and the speech and language progressed gradually and continuously until about age 5 years. All of the children that were evaluated used vocalization to attract attention and to communicate.

The MAIS (Meaningful Auditory Integration Scale) is designed to identify the meaning of hearing loss for a child that uses sound in daily life (Robins et al., 1991).^14^

The IT-MAIS is used for identifying hearing abilities in very young children; it surveys spontaneous auditory behaviors that children present in daily living, using examples in three different hearing ability developmental areas.

These three areas include vocalization changes associated with using the device, alertness to environmental sounds and attribution of meaning to sounds. Using information provided by parents, an examiner scores each question, according to the occurrence frequency of the behavior, from 0 (“never showed this behavior”) to 4 (“always showed this behavior). The maximum IT-MAIS score is 40 (Robins et al., 2003).^15^

In our clinical work we have found that the parents's description is out of phase with our observation of their child's development and their use of individual sound amplification devices. It is important to point out that objective methods have been preferred in the literature; these usually are language and speech perception evaluation protocols that provide quantitative measurements of performance, as shown in studies by Bevilacqua et al. (1997),^9^ Pulsifer et al. (2003),^8^ Myamoto et al. (2003),^6^ and Ouellet et al. (2001).^11^

## OBJECTIVE

This study aimed to compare video recorded results of interaction situations of cochlear implant candidates with results of evaluation protocols based on parent responses, aiming to reflect about the validity of these procedures in assessing children considered as candidates for cochlear implants.

## MATERIAL AND METHOD

### Definition of the sample

The sample included profoundly deaf children that had been accepted by the Cochlear Implant Sector of a hospital in the Sao Paulo state hinterland according to the following criteria:

1 profound bilateral sensorineural hearing loss;
2 at least three months experience with a hearing aid;
3 inability in closed-set word recognition;
4 adequate and motivated family for using a cochlear implant;
5 adequate rehabilitation conditions in the city of origin;
6 age below 3 years and 11 months at the time of inclusion into the program.

The cochlear implant medical service chosen for this study undertakes on average four such surgeries in children each month. Fifty percent (eight children) of those children selected in the second semester of 2004 (August to November) were invited to participate in this study. Family members received an information letter describing the research procedures; the children were included in the study only after parents approved participation and signed a free informed consent form. Children whose participation was not authorized were not included.

The final sample included seven children, three female and four male. The mean age was 39.7 months, ranging from 25 months to 52 months.

**Chart 1:**
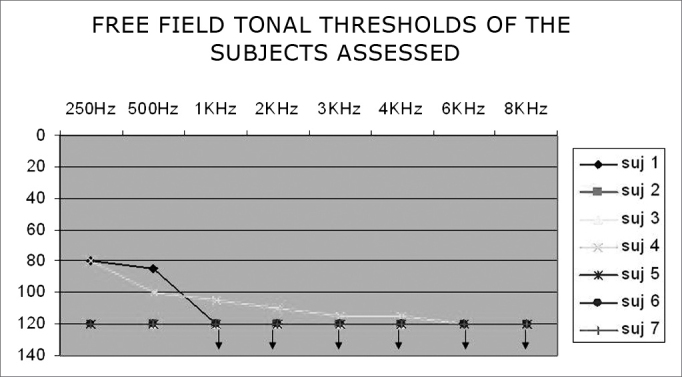
Free-field pure tone thresholds of subjects - Note: Observe response overlap in subjects 2, 3, 5, 6 and 7.

Free-field pure tone audiometric thresholds of the subjects may be seen on Chart 1.

**Chart 2:**
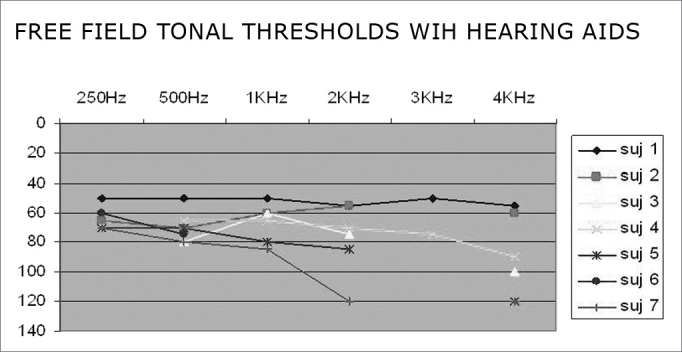
Free-field pure tone thresholds with hearing aids - subjects 1, 2, 3, 4, 5, 6 and 7 - subjects of the 0 – 140 dB sample - Free field pure tone thresholds with hearing aids.

Chart 2 shows free-field pure tone thresholds when using hearing aids.

**Chart 3:**
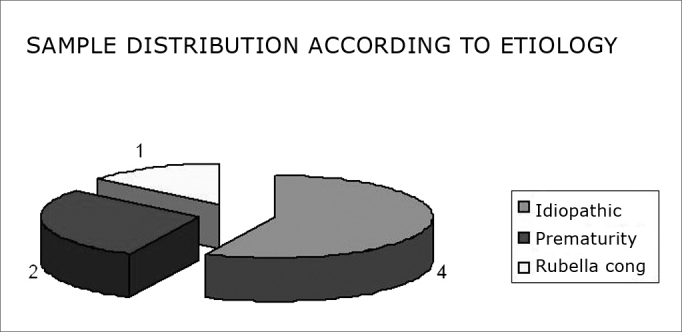
Distribution of the sample according to the etiology - subjects of the sample distributed according to the etiology of hearing loss.

All of the study subjects had congenital hearing loss. The etiology of hearing loss is shown in Chart 3.

Brainstem evoked auditory potentials, and transient and distortion product evoked otoacoustic emissions were absent in all of the children included in the sample.

### Procedure

All of the children underwent an evaluation done by the Cochlear Implant Sector for selecting the best candidates for cochlear implants. IT- MAIS (Infant Toddler - Meaningful Auditory Integration Scale, Castiquini, 1998)^16^ and MUSS (Meaningful Use of Speech Scale, Nascimento and Bevilacqua, 1997)^13^ questionnaire data were assessed. These questionnaires are applied by speech therapists as part of language evaluation procedures.

The IT-MAIS is an open and closed-question questionnaire that aims to assess auditory speech perception. Points are given for each answer, resulting in a score according to the following questionnaire categories:

### Category 0 - speech not detected

This child does not detect speech in normal conversation conditions (speech detection threshold > 65 dB).

### Category 1 - detected

This child detects speech signs.

### Category 2 - perception pattern

This child differentiates between words by suprasegmental parameters (duration, tonicity, etc.). For ex.: hand X show (mão x mostrar), house X boy (casa x menino)

### Category 3 - initial word identification

This child differentiates between closed-set words based on phonetic information. This pattern may be demonstrated with words of equal duration but multiple spectral differences. For ex.: fridge X bike (geladeira x bicicleta), cat X house (gato x casa).

### Category 4 - word identification by vowel recognition

This child differentiates between closed-set words that primordially differ in the vowel sound. For ex.: foot, dust, spade (pé, pó, pá); hand, mine, me (mão, meu, mim).

### Category 5 - word identification by consonant recognition

This child differentiates between closed-set words with the same vowel sound, but differences in consonants. For ex.: hand, bread, so, dog, floor (mão, pão, tão, cão, chão).

### Category 6 - open-set word recognition

This child is able to hear words out of context and extract sufficient phoneme information to recognize a word solely by listening to it.

MUSS is a closed question questionnaire aimed at assessing oral language use in children. Like IT-MAIS, points are given to fit a child into the following categories:

### Category 1

This child does not speak and may present undifferentiated vocalization.

### Category 2

This child speaks isolated words.

### Category 3

This child builds two to three element phrases.

### Category 4

his child builds 4 or 5 word phrases and begins to use connecting elements.

### Category 5

This child builds phrases with over five words, uses connecting elements, conjugates verbs, uses plurals, etc. The child is fluent in oral language.

The sample children were videotaped while in non-directed play; this was done in an empty room. Two boxes containing toys (saucepans, wild animals, domestic animals, cars, trains, modeling clay, paper, pencils, masks, etc.) were presented to each child, and the mother or father was asked to play with the child during video recording; the evaluator participated only if invited by the child. Filming was done for about 20–30 minutes, aiming to observe interactions that might bring information about the children's language abilities. The videos were observed at a later moment, and excerpts of interest to this study were selected; in these, the children's behavior was observer and classified into the following analysis categories:

– use of speech
– speech understanding
– use of onomatopoeias
– use of gestures
– gesture understanding
– communicative intention
– presence of turns
– symbolic games

Various levels of complexity in child behavior were taken into account, as shown on Table 2. The symbol + was attributed according to the complexity of each datum/segment observed within each of the abovementioned categories. For the category speech, + was attributed to children who used isolated words only; in speech understanding, + was attributed to children who understood isolated words within a context, with help from lip reading, ++ for children who understood two-word phrases with help from lip reading; in use of onomatopoeias, + was attributed when onomatopoeias occurred. For use of gesture, + was attributed when children used only indicative gestures, ++ for children who used representative gestures, and +++ for children who used Brazilian sign language; for gesture understanding, + was attributed to children who understood only isolated concepts/gestures within a context; ++ was attributed when children used supportive gestures to partially understand aspects of a given context, and +++ when children demonstrated that they predominantly understood the gestures made by interlocutors. For the item communicative intention, + was attributed when children showed little interest in communicating, ++ when children sought others for a few communicative exchanges, and +++ when a communicative intention predominated during an interaction. For presence of turns, + was attributed when there was sporadic alternation of discursive turns, ++ when this alternation was partial, and +++ when alternation of discursive turns predominated during the interaction. Finally, in the item symbolic games, the number of +s varied according to the degree of game elaboration; +++ was only attributed to children that demonstrated gaming with a functional action that indicated further elaboration, and therefore, more structured language.

**Table 2:** Language aspects seen during video recording and the results for each MUSS and IT-MAIS questionnaire category:

	speech	Speech understanding	Onomatopoeias	Use of gestures	Gesture understanding	Communicative intention	Presence of turns	Symbolic games	IT-MAIS	MUSS
Subj.1	+	++	+	+	+	+++	+++	++	1	2
Subj.2			+	++	+++	+++	+++	+++	0	1
Subj.3		+	+	+	+	+++	++	++	0	1
Subj.4	+	+	+	+++	+++	+++	+++	++	1	2
Subj.5			+	++	+	++	+	+	0	1
Subj.6				+	+	++	++	+	0	1
Subj.7		+		++	++	+++	+++	++	0	1

**Key:**

Speech: + was attributed to children that used isolated words only.

Speech understanding: + was attributed to children who understood isolated words within a context, with help from lip reading; ++ for children who understood two-word phrases with help from lip reading.

Onomatopoeias: + was attributed when onomatopoeias occurred.

Use of gestures: + was attributed when children used only indicative gestures, ++ for children who used representative gestures, and +++ for children who used Brazilian sign language.

Gesture understanding: + was attributed to children who understood only isolated concepts/gestures within a context; ++ was attributed when children used supportive gestures to partially understand aspects of a given context, and +++ when children demonstrated that they predominantly understood the gestures made by interlocutors.

Communicative intention: + was attributed when children showed little interest in communicating, ++ when children sought others for a few communicative exchanges, and +++ when a communicative intention predominated during an interaction.

Presence of turns: + was attributed when there was sporadic alternation of discursive turns, ++ when this alternation was partial, and +++ when alternation of discursive turns predominated during the interaction.

Symbolic games: the number of +s varied according to the degree of game elaboration; +++ was only attributed to children that demonstrated gaming with a functional action that indicated further elaboration, and therefore, more structured language.

It is important to state that absence of a “+” in any of these categories reflects absence of that behavior or that it was not observed during video recordings.

MUSS and IT-MAIS evaluation protocol results were paired qualitatively with the video session of each child, and the results were presented individually.

The Research Ethics Committee approved this study (protocol 554/2003).

## RESULTS

Table 1 shows MUSS and IT-MAIS results for each child:

**Table 1:** Distribution of sample subjects according to MUSS and IT-MAIS questionnaire categories:

	MUSS	IT-MAIS
Subj 1	2	1
Subj. 2	1	0
Subj. 3	1	0
Subj.4	2	1
Subj.5	1	0
Subj.6	1	0
Subj.7	1	0

Five of seven children were classified in IT-MAIS category 0, that is, they are incapable of detecting the presence of speech even when using hearing aids; two were classified in category 1, indicating that they only perceived speech signs, but were incapable of perceiving suprasegmental parameters or of discriminating any words. These results were expected, as these were children whose caretakers had sought a cochlear implant program, which benefits subjects that had poor responses with hearing aids.

Five or seven children were classified in the MUSS questionnaire as language category 1, indicating that these children did not speak or issued only undifferentiated vocalizations; two of the children were classified as language category 2, indicating that these children spoke only isolated words.

Table 2 shows the evaluation results of language aspects observed in video recordings of the children in non-directed play situations, paired with questionnaire results.

Video recordings showed significant heterogeneicity between the children, and provided important information that had not become evident in the questionnaires. We were able to see that only two children used words isolatedly, that only three children understood speech with support from lip reading, and that five children used onomatopoeias. All children used gestures, but only one child used Brazilian sign language; all children understood gestures, but only one of them predominantly used gestures for support. Most of the children showed good communicative intention and respected turns. All children had some level of symbolic gaming, but only one showed gaming with functional action.

## DISCUSSION

Table 2 shows that children classified in the same MUSS and IT-MAIS questionnaire categories presented very different behaviors when observed at play, and are, therefore, not in the same group as the questionnaires might have us believe. This table reveals that, in relation to forms of communication, all children that had speech used only words. The same occurred in relation to speech understanding; most of the children that understood speech did so using lip reading only of isolated words that belonged to the context. All children used gestures in various manners: indicative gestures, representative gestures, nodding, shaking their head; some children used part of the Brazilian sign language. These results are significantly different from the MUSS questionnaire results for questions about the use of gestures, most of which reported absence of gestures.

Some children understood gestures isolatedly in interactions. Once again MUSS questionnaire answers mostly indicated that children understood little about gesture use.

We underline that when parents arrive at the cochlear implant service we analyzed, the first contact is made by a social worker who provides information about candidate selection criteria and explains, among other things, that the program prefers candidates that have received oral stimulus. We believe, then, that parents may deny gesture use and understanding, in an attempt to increase the possibilities that their child will be selected.

Communicative intention was analyzed by taking into account children's interest in oral communication or in any form of language expression indicating a wish to communicate. For instance, in contact with toys, children were not content only to explore them, but rather, they sought their interlocutors to show that they knew or not something about that object. It is essential for children to express their desire to communicate; children reconstruct internally cultural forms of action, thought and meaning based on these relationships with others (Roncato and Lacerda, 2005).^17^

Respect for others in oral or non-oral dialogue was also analyzed. This may be seen when children respect and/or express themselves according to the propositions presented by their interlocutors.

Symbolic gaming was also noted, considering that it assumed action schemes (the child acting on the world) and interaction schemes (the child in necessary collaboration and sharing with others) (Scarpa, 2000).^18^ In this category, we attempted to see if children used objects functionally or only for exploration purposes; or if they played in context, and if they understood rules set by the interlocutor (for example: blowing on food before offering it to each doll that were members of a family). Games with rules involve content and preestablished action to set limits for the child's activity, according to Kishimoto (2004).^19^ Respect for categorization was also noted (for example: separating pets from wild animals, or grouping all of the cars within a fence). We checked to see if children's play was organized, or if children gave clues so that interlocutors for them to interact and learn the aim of a given game. We believe that children's worlds are organized based on symbols that generate representative levels favoring the emergence of conditions for language acquisition, as contended by Lacerda (2004).^20^

Use of onomatopoeias was also noted in interactions (for example: use of the onomatopoeia “au-au” in referring to dogs). We considered “use” when there was not only repetition or imitation, but also when the onomatopoeia had representation and communication functions. Onomatopoeias report to sounds that animals produce and the manner by which each culture reproduces that sound, which indicates probable use of hearing in children.

It was possible to observer data compatible with each auditory phase by comparing the questionnaire responses and the analysis of children at play. Responses given by mothers in the questionnaires are comparable with the children's auditory behavior seen when at play, considering that these are profoundly hearing-impaired children (a condition for using cochlear implants). The MAIS scale has been used in many studies to assess cochlear implant use in daily activities (Allum et al., 2000;^21^ Nikolopoulos et al., 2005;^22^ Weichbold et al., 2004^23^). Bosco et al. (2005)^24^ demonstrated the efficacy of this tool in revealing performance differences between two groups of implant users in which the implants were equal but with different resolutions. The MAIS confirmed what had been demonstrated in the test battery used for assessing speech perception. Robins et al. (2003)^15^ used the IT-MAIS successfully to check the progression of auditory behavior in children aged below three years that had implants.

In language issues, questionnaire data differ significantly from data observed in play sessions. Analysis of questionnaires placed children into oral language use categories. Although questionnaires also include questions about communicative intention, use of gestures and communication strategies, these aspects are not effectively taken into account for categorizing purposes. Thus, the questionnaire grouped children into only two categories (shown on Chart 4). In observing these children at play, however, we found that children included in a similar category by the questionnaire had markedly different linguistic behaviors, as shown on Table 2.

The questionnaires take into account only the use of oral language, in which most of the sample children scored poorly, as expected for young profoundly hearing-impaired children not using hearing aids. Our question, then, is: what is the purpose of applying such a questionnaire if answers are inherent to the state of the child being assessed, a state required for acceptance into the cochlear implant program?

Allum et al. (2000)^21^ used protocols to evaluate the performance of cochlear implant child users; the MUSS questionnaire results were not shown, as these authors believe that this tool is insufficient for demonstrating significant improvements in linguistic behavior. The same happens in Bosco et al.'s 2005^24^ study.

We believe that conditions for evaluating the linguistic behavior and language-related aspects are improved by taking into account the observation of children at play. These factors may help define the post-implant progression in greater detail, compared to the MUSS questionnaire categories. Nikopoulos et al. (2005)^22^ have stated that video recording is ideal for observing the behavior of pre-verbal communication in deaf children, responses in interactions with known adults, eye contact, respect for turns, voice initiative, gesture initiative, and auditory knowledge.

Appropriate selection of child candidates for cochlear implants is essential for an improved prognosis. It is, therefore, inefficient for the selection process to focus the evaluation on closed protocols that provide recurrent and already-known data about profound hearing-impaired children. Assessing the communicative intention and the forms by which these children interact with others could be more important in choosing the best candidates for cochlear implants. A child develops as a subject of language in social interaction, in the manner by which interlocutors allow us to speak and consider us as speaking beings (Santana, 2005).^25^

## CONCLUSION

The results of auditory assessment protocols (IT-MAIS) were compatible with the results found by observing play, indicating that playing adequately expresses the true auditory condition of children.

The results of oral language protocols were significantly different from those found by observing children in play. One of the reasons for this might be that these results reflect caretakers's views about children - affected not only due to emotional involvement, but also by a concern in providing answers that might facilitate children being accepted in the program - which would distort reality.

The results suggest that a child's communicative intention, behavior in dialogical situations, and forms of seeking interaction with others, may all be observed in play. Furthermore, play also provides ample information about their language development level and their reaction to communication situations when using cochlear implants. These data are even more effective and realistic when evaluating children's attitude about communication and interaction with others; this may be of significant interest when assessing candidates for cochlear implants.
